# Identifying the Genetic Basis of Fetal Loss in Cows and Heifers Through a Genome-Wide Association Analysis

**DOI:** 10.3390/ani16020293

**Published:** 2026-01-17

**Authors:** Ousseini Issaka Salia, Emaly M. Suarez, Brenda M. Murdoch, Victoria C. Kelson, Allison L. Herrick, Jennifer N. Kiser, Holly L. Neibergs

**Affiliations:** 1 Department of Animal Science, Washington State University, Pullman, WA 99164, USA; o.issakasalia@wsu.edu (O.I.S.);; 2 Department of Animal, Veterinary and Food Sciences, University of Idaho, Moscow, ID 83844, USA; bmurdoch@uidaho.edu; 3 Washington Animal Disease Diagnostics Laboratory, Pullman, WA 99164, USA; jennifer.kiser@wsu.edu

**Keywords:** dairy, primiparous cows, heifers, genome-wide association analysis, loci, fetal loss

## Abstract

Fetal loss, the spontaneous termination of pregnancy between day 42 and 260 of gestation, is poorly understood. Impacts of fetal loss include loss of production, increased health risk, and economic loss. The aims of this study were to identify loci associated with fetal loss in Holstein heifers and primiparous cows to facilitate the selection of reproductively efficient cattle and identify the genetic causes of fetal loss. A genome-wide association analysis (GWAA) compared 5714 heifers that calved at term (controls) to 416 heifers that experienced fetal loss (cases), and for primiparous cows, 2519 controls were compared to 273 cases. The efficient mixed-model association eXpedited approach in the SNP and Variation Suite (v 9.1) statistical software was used with additive, dominant, and recessive inheritance models for the GWAA. In heifers, 16 loci were associated (FDR < 0.05) with fetal loss in the recessive model. In primiparous cows, there were 44 loci associated (FDR < 0.05) with fetal loss in the recessive model. No loci associated with fetal loss were shared between cows and heifers or in the additive and dominant models. These results improve the characterization of genetic factors contributing to fetal loss in Holstein heifers and primiparous cows and provide targets for genomic selection.

## Simple Summary

Fetal loss in cattle is defined as a pregnancy loss between day 42 and day 260 of gestation, after which the calf can survive outside of the uterus. Fetal loss negatively impacts cattle health and productivity, and results in substantial economic losses to the dairy industry. This study aimed to better understand the genetic causes of fetal loss in dairy heifers (a female being bred for her first pregnancy) and primiparous cows (a female in her first lactation being bred for a second pregnancy). To account for multiple testing in this genome-wide association analysis (GWAA), associations were evaluated using the false discovery rate (FDR), which represents the expected proportion of false positives among all rejected hypotheses. A total of 16 genomic regions and 27 positional candidate genes were identified as associated (FDR < 0.05) with fetal loss in heifers, and 44 regions and 87 positional candidate genes were identified as associated (FDR < 0.05) with fetal loss in primiparous cows. The identification of these genomic regions and genes associated with fetal loss facilitates selection for cattle that are more reproductively efficient and provides a better understanding of the causes of fetal loss.

## 1. Introduction

Gestation in cattle averages approximately 280 days [1,2,3]. Pregnancy loss is typically categorized by gestational age as it correlates with development stages of the embryo and fetus, with losses before day 42 considered embryonic and those occurring between days 43 and 260 classified as fetal [1,4,5,6]. Fetal loss is less common than embryonic loss and varies by parity [7,8]. Embryonic loss is the most prevalent form of reproductive failure in dairy cattle, accounting for approximately 25–40% of pregnancies that fail to result in a live calf [5,8,9,10]. In contrast, fetal loss occurs at lower frequencies but varies substantially by parity, with reported rates ranging from 2 to 12% in heifers and 4 to 24% in primiparous cows, occasionally exceeding these values in certain herds [5,7,8,11,12,13]. Revenue in the dairy cattle industry directly depends on reproductive efficiency, as the production of a calf is required for lactation [14]. Higher reproductive rates lead to fewer inseminations, lower costs associated with insemination, and shorter intervals between calving and subsequent lactations [15,16].

Fertility traits, including pregnancy maintenance, are complex and influenced by both genetic and non-genetic (environmental, physiological) factors [17,18,19,20,21]. While many factors, including inadequate nutrition, stress, housing conditions, parity, and infectious disease, can impact fertility [17,18,20,22], genetic variation also plays an important role in both embryonic and fetal survival [23,24].

Recent genomic studies have revealed that fetal loss is influenced by multiple genes [25,26,27,28,29]. Heritability represents the proportion of phenotypic variation explained by additive genetic variation within a population [30,31] and reflects the potential for genetic improvement through selection [32]. Reported heritability estimates for fetal loss vary from 0.01 to 0.18 across parities [7,12,29] and indicate that although non-genetic factors play a substantial role, genetic variation contributes to differences in pregnancy maintenance among females [7,32]. These heritability estimates indicate that consistent selection to reduce fetal loss will improve successful pregnancy maintenance [7].

The genetic basis for fetal loss in Holstein heifers and primiparous cows is not well understood. In smaller studies, fetal loss has been associated with loci in recessive inheritance models, which is consistent with loss of fertility, calving difficulties, and embryonic mortality identified in inbred lines of cattle [33,34,35]. Loss of fertility in inbred lines is believed to be due to the concentration of homozygous genotypes associated with inbreeding [36]. The increase in homozygosity leads to a lack of fitness in cattle, defined by the ability to survive and reproduce [36]. For the study of a comprehensive evaluation of the genetic basis of fetal loss, it is therefore imperative to consider the recessive inheritance model, as well as the more commonly used additive model needed for heritability estimates, and the dominant inheritance model.

To understand the genetic foundation of fetal loss, and to aid in selecting and managing dairy heifers and cows for a breeding herd that will be reproductively efficient and profitable, a genome-wide association analysis (GWAA) approach was used. This is a powerful method to identify the genetic basis of complex traits [37,38], including pregnancy loss [12,28,29,39,40,41]. Therefore, the aim of this study was to identify loci and positional candidate genes associated with fetal loss in Holstein heifers and primiparous cows using a GWAA with additive, dominant, and recessive inheritance models.

## 2. Materials and Methods

### 2.1. Study Population and Phenotypes

A single dairy in Georgia provided access to genotypes and performance records for 20,344 nulliparous heifers and 10,584 primiparous cows. This study was approved by the Washington State University Institutional Animal Care and Use Committee #6743. Pregnancy was determined by ultrasound at approximately 30 days post breeding. Only Holstein heifers (*n* = 6,314) and primiparous cows (*n* = 2,882) that conceived after the first artificial insemination (AI) service were used for this study to remove possible confounding of the phenotype for embryonic loss. Heifers and primiparous cows were divided into case and control groups. Controls were those cattle that maintained their pregnancy > 260 days, and cases were those cattle that had fetal loss between gestational days 42 and 260.

Health and breeding records were obtained using Dairy Comp 305 (Valley Agricultural Software, Tulare, CA, USA) to identify animals who experienced fetal loss after being bred by artificial insemination (AI). Data for breeding, production, and disease were recorded daily. The frequency of fetal loss was investigated based on the sires that heifers and cows were bred to. There were 114 sires bred to heifers and 117 sires bred to cows.

To emphasize genetic factors associated with fetal loss, heifers (*n* = 164) and primiparous cows (*n* = 66) were removed from the study if they experienced mastitis, metritis, metabolic disease, respiratory disease, or lameness at any time during their pregnancy, as these factors are known to contribute to infertility [42,43,44,45,46]. Additionally, four heifers and two primiparous cows were excluded due to death or being culled prior to calving. Furthermore, animals were excluded that were placed on synchronization protocols that involved fewer than 10 animals to reduce potential bias, resulting in the removal of 16 heifers and 22 primiparous cows from the analysis. After quality control, there remained 5714 control and 416 cases for the heifer GWAA and 2519 controls and 273 cases for the primiparous cow GWAA.

### 2.2. Genotyping and Imputation

Cattle were genotyped by STGenetics (Navasota, TX, USA) using their proprietary SNP panel of approximately 70,000 single-nucleotide polymorphisms (SNPs). SNP alleles with minor allele frequency (MAF) < 0.01 were removed prior to imputation to a higher density of approximately 634,321 SNPs based on the Illumina BovineHD BeadChip (San Diego, CA, USA). Imputation was performed with Beagle (v. 4.1) [47] using a Holstein reference population and an accuracy of 95% as previously described [28]. Imputation accuracy was determined by genotyping animals in the reference population at high density (approximately 778,000 SNPs) with the Illumina BovineHD BeadChip, and then removing SNPs to a total of approximately 50,000 SNPs. Imputation was then performed on the removed SNPs up to the 778,000 SNP level to calculate the accuracy of imputation. Genotypes from the imputed SNPs were compared with the SNPs that were called from the initial genotyping with the 50,000 SNPs (BovineHD BeadChip).

### 2.3. Genotyping Quality Control

The 634,321 imputed SNPs were subjected to quality control before the GWAA. In the dataset for heifers, SNPs with a call rate < 90% (278 SNPs), or those with a minor allele frequency < 1% (53,523 SNPS) were removed using VCF tools version 0.1.16 [48]. No SNPs were removed due to having a call rate < 90%, but 53,101 SNPs were removed due to a minor allele frequency < 1% in the primiparous cows. Hardy–Weinberg equilibrium testing was used to identify and remove 21,297 SNPs that failed (*p* < 1 × 10^−25^) in heifers and 11,914 SNPs that failed (*p* < 1 × 10^−20^) in primiparous cows using PLINK (version 1.9) [49]. Significance thresholds for Hardy–Weinberg equilibrium testing were identified after plotting and identifying the SNPs that comprised the extreme tails of the distribution. No animals were removed for >10% of their genotypes failing to be called [49]. Following quality control filtering, a total of 6130 heifers were evaluated with 559,223 SNPs and 2792 primiparous cows were evaluated with 570,306 SNPs for the GWAA.

### 2.4. Genome-Wide Association Analysis

A GWAA was conducted using SNP and Variation Suite (SVS) software version 9.1 (Golden Helix, Bozeman, MT, USA) to identify loci associated with fetal loss in Holstein heifers and primiparous cows. The analysis used the efficient mixed-model association eXpedited (EMMAX) method in SVS, incorporating an identity-by-state matrix [50]. The general mixed model used in EMMAX is defined as *γ* = *Xβ* + *Zµ* + *ε*, where *γ* represents the vector of observed phenotypic values; *X* is the matrix of fixed effects; β denotes the regression coefficients; *Z* is the matrix of observed random effects; *µ* is the vector of random effects related to allele substitution variants in the population; and *ε* represents the residuals effects [50].

Three GWAA models (additive, dominant, and recessive inheritance) were applied to determine the genetic basis of fetal loss in Holstein heifers and primiparous cows [28,29]. In the additive model, it was assumed that individuals with two minor alleles (aa) have twice the effect on fetal loss as those with a single minor allele (Aa). The dominant model assessed the association by comparing genotypes (AA and Aa) against (aa), while the recessive model compared (AA) with the combined group of (Aa and aa) genotypes [25,51,52].

A false discovery rate (FDR) correction was applied in SVS to account for multiple testing, and associations with fetal loss were considered significant when FDR < 0.05 [53]. Linkage disequilibrium (LD) between loci was evaluated using the standardized disequilibrium coefficient D’, as described by Lewontin (1964) [54]. Pairwise D’ values were calculated for SNPs located on the same chromosome that were significantly associated with fetal loss. D’ > 0.7 was used to define locus boundaries when SNPs on the same chromosome were associated with fetal loss. This threshold has been previously used to characterize loci in both humans and cattle [25,28,29,55,56,57]. This cutoff of D’ > 0.7 represents strong LD and is consistent with empirical LD patterns reported in the dairy cattle population. In North American Holsteins, an average D’ value of approximately 0.72 was observed for SNPs by 40–60 kb, indicating substantial LD across the genome [57]. Therefore, a threshold of D’> 0.7 was considered biologically appropriate for defining loci in the present study.

### 2.5. Identification of Population Stratification

To assess population stratification, a principal component analysis (PCA) was performed for the Holstein heifer and primiparous cow populations (Figure S1A,B). To identify sources of population structure, the effects of covariates such as AI (artificial insemination) sires, AI technicians, synchronization protocols, and animals’ birth year and conception month on fetal loss were evaluated using ANOVA in R (v. 4.5.1) [58]. The ANOVA model used was as follows: Y_ijklmn_ = μ + Sire_i_ + BirthYear_j_ + ConceptionMonth_k_ + Protocol_l_ + Technician_m_ + (Sire × Protocol)_il_ + (Sire × BirthYear)_ij_ + (BirthYear)_jl_ + ε_ijklmn_, where Y_ijklm_ is the fetal loss phenotype of the nth animal, μ is the grand mean, Sire is the effect of the ith AI sire, BirthYear is the effect of the jth birth year, ConceptionMonth is the effect of the kth conception month, Protocol is the effect of the lth synchronization protocol, Technician is the effect of the mth AI technician, and the interaction terms account for biologically relevant dependencies between factors. The value ε_ijklmn_ is the residual error associated with each animal. Only those with significant effects on fetal loss (*p* < 0.05) were included as fixed effects in the GWAA for the corresponding population (Holstein heifers or primiparous cows). There was a difference in fetal loss by AI sire in Holstein heifers (*p* < 0.05) and primiparous cows (*p* < 0.05). The effects of AI technician and synchronization protocols on fetal loss were not significant in either population (*p* > 0.05). Birth year significantly affected fetal loss in Holstein heifers (*p* < 0.05) but not in primiparous cows (*p* > 0.05). Conversely, conception month had a significant effect on fetal loss in primiparous cows (*p* < 0.05) but not in Holstein heifers (*p* > 0.05). Covariates with a significant effect on fetal loss (*p* < 0.05) were accounted for in the GWAA for the respective affected population. Residual population stratification after covariate correction was assessed using the genomic inflation factor (λ_GC_), following the method of Devlin and Roeder (1999) [59,60]. The λ_GC_ values were close to 1.0 across additive, dominant, and recessive inheritance models in both populations (Holstein heifers: 1.0, 0.98, 1.0; primiparous cows: 1.0, 1.0, 0.99), indicating minimal inflation and negligible residual stratification. The consistency of λGC values across multiple inheritance models supports the robustness of the association results.

### 2.6. Proportion of Variance Explained

The proportion of variance explained by an SNP was calculated in SVS using the notation of Further Optimization When Covariates Are Present [61,62]. As SNPs within a locus are not independent due to LD, the sum of the proportion of variance explained for all SNPs exceeds 100%.

### 2.7. Estimation of Heritability

The heritability of fetal loss in Holstein heifers and primiparous cows was estimated using the best genomic linear unbiased predictor (GBLUP) method [63,64], implemented with the average information algorithm (AI-REML), a bivariate restricted maximum likelihood approach [65]. In this method, AI-REML GBLUP estimates variance components, which are then used to calculate heritability [63,64], providing a more accurate estimate than the pseudo-heritability from EMMAX [50], which can be inflated in studies with limited sample sizes.

### 2.8. Positional Candidate Genes

To identify positional candidate genes within a locus and compare loci associated with fertility and production traits in previous studies, the average haplotype was estimated for this population in SVS using the method of Gabriel et al. (2002) [66]. An average haplotype size of 29 kb was estimated. Positional candidate genes were genes located within 29 kb of the 5′ and 3′ of the SNP associated with fetal loss using the bovine genome assembly ARS-UCD2.0 (https://www.ncbi.nlm.nih.gov/datasets/genome/) (accessed on 6 June 2025).

### 2.9. Loci Reported in Previous Fertility Studies

Loci previously identified as associated with fertility were compared to loci identified as associated with fetal loss in this study using the literature and searches in the animal QTL database (http://www.animalgenome.org/QTLdb accessed on 6 June 2025) [67,68]. If the associated SNPs from the previous study and the current study were within 29 kb (a haplotype) of one another, the loci were considered to be shared. Targeted fertility traits included spontaneous abortion [28,29], conception rate [69,70,71], number of times bred [70], daughter pregnancy rate [72], number of inseminations per conception [73], days from calving to first insemination [73], and fertility index [73].

### 2.10. Comparison of Loci Associated with Fetal Loss and Production Traits

To identify shared loci that may suggest genetic interactions influencing fertility, loci associated with fetal loss in Holstein heifers and primiparous cows were compared to loci associated with traits related to milk production such as milk yield, milk protein yield, milk fat yield, and milk fatty acid amount using the literature and searches in the animal QTL database (http://www.animalgenome.org/QTLdb) (accessed on 6 June 2025) [67,68]. Phenotypic correlations of cattle experiencing fetal loss and milk production traits were not possible in heifers as cattle with fetal loss are frequently culled.

## 3. Results and Discussion

### 3.1. Loci Associated with Fetal Loss in Heifers

**Figure 1:**
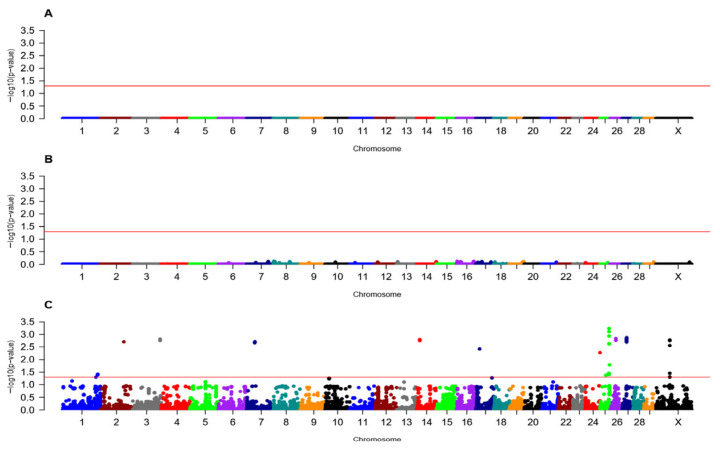
Loci associated with fetal loss in Holstein heifers in additive (**A**), dominant (**B**), and recessive (**C**) inheritance models. The X-axis shows the 30 chromosomes (1 to 29 and X) by colors, representing the physical positions of each SNP, while the Y-axis displays the −log10FDR value for each SNP. The solid red line represents the significance threshold for association (FDR < 0.05) with fetal loss in Holstein heifers. The Y chromosome is not shown since all animals in the study were female.

In heifers, 35 SNPs across 16 loci were associated (FDR < 0.05) with fetal loss in the recessive inheritance model, but no loci were associated in the additive or dominant inheritance models (Figure 1, Table 1 and Table S1). Similar GWAA results were reported by Suarez et al. (2024) [28], where most of the associated loci were detected under the recessive model in their study on genomic regions associated with spontaneous abortion in Holstein heifers [28]. For the recessive model, loci associated with fetal loss were on chromosomes 1, 2, 3, 7, 14, 24, 25, 26, 27, and X. BTA25 contained the highest number of associated loci (four) and SNPs (12) and the SNP with the greatest significance (FDR = 5.80 × 10^−4^, Figure 1, Table S1). The 16 loci individually explained between 0.003% and 0.005% of the additive genetic variance (Table 1). Twenty-seven positional candidate genes were identified in association with fetal loss in Holstein heifers (Table 1 and Table S1), with the highest number of associated positional candidate genes (*CNNM2*, *TAF5*, *ATP5MK*, *MIR1307*, *PDCD11*, and *LOC104975977*) within locus 13 on BTA26 (Table 1 and Table S1). Positional candidate genes were not identified for locus 1 on BTA1, locus 3 on BTA2, locus 4 on BTA3, locus 6 on BTA14, locus 7 on BTA17, locus 9 on BTA25, or locus 14 on BTA27 (Table 1).

In Holstein heifers, among the three most significant loci associated with fetal loss (locus 10 on BTA25, locus 15 on BTA27, and locus 13 on BTA25), all contained positional candidate genes. Four positional candidate genes (*TMEM225B*, *LOC112444278*, *ZNF655*, and *ZNF789*) were associated with the most significant locus (FDR = 5.8 × 10^−4^) on BTA25. These positional candidate genes were previously identified as positional candidate genes for spontaneous abortion in AI-bred Holstein heifers [28]. As this is the second study in an independent population of Holstein cattle that has identified this association with fetal loss, this locus warrants consideration for use in genomic selection for fertility in heifers. *TMEM225B*, *ZNF655*, and *ZNF789* have also previously been reported as having physiological roles in fertility.

Transmembrane protein 225B (*TMEM225B*) on BTA25 is also known to be a testis-specific transmembrane protein which plays an important role in male fertility by ensuring proper sperm maturation [74]. Functional studies have shown transmembrane proteins to be expressed during pregnancy [75]. Interestingly, other transmembrane proteins from the *TMEM225B* family (*TMEM120A*, *TMEM189*, *TMEM50B*, and *TMEM9*) were expressed in day 17 conceptus and in the placental chorion on gestational days 24, 30, and 50 in cattle when evaluated by single-cell data [75].

Zinc finger proteins constitute one of the largest and most diverse families of transcriptional regulators, characterized by their ability to bind not only DNA but also proteins, lipids, and poly-ADP-ribose, which allow them to be involved in a wide range of cellular functions [76]. Zinc finger proteins were also found to be associated with immune system regulation at both transcriptional and post-transcriptional levels [76]. Besides the association with fetal loss in our study and with spontaneous abortion in Holstein heifers [28], zinc finger protein 789 (*ZNF789*) was associated with infertility in women diagnosed with endometriosis [77]. A polymorphism (*rs259983*) in other zinc finger proteins (*ZNF831*) increased the risk of superimposed preeclampsia in women with gestational diabetes mellitus [78]. Based on these findings, zinc finger protein may play an important role in reproduction by influencing gene expression, immune function, and pregnancy maintenance.

*ZNF655*, also within locus 10 on BTA25, is associated with fetal loss in Holstein heifers. *ZNF655* interacts with the integrator complex in transcription regulation and RNA processing [79,80]. Given this role, and the consequences of aberrant regulation and processing of transcription, it is plausible that *ZNF655* would be detrimental to a developing fetus. This gene is also targeted by miRNA during the first trimester in peripheral serum of women with gestational diabetes mellitus [81].

None of the positional candidate genes (*LOC112444679*, *LOC107131907*, and *LOC781220*) in the second most significant locus (FDR = 1.34 × 10^−3^) on BTA27 had functions known to be related to fertility. The third most significant locus (FDR = 1.44 × 10^−3^) on BTA26 contained six positional candidate genes (*CNNM2*, *TAF5*, *ATP5MK*, *MIR1307*, *PDCD11*, and *LOC104975977*). Of these positional candidate genes, *MIR1307* and *PDCD11* have functions that are most closely aligned with fetal loss.

MicroRNAs (miRNAs) such as *MIR1307* on BTA26 play an important role in female fertility by modulating ovarian function, endometrial receptivity, and embryo–maternal communication [82,83]. Abnormal miRNA may affect cellular proliferation, migration, and progesterone responsiveness, which can disrupt implantation and lead to infertility [84]. MicroRNA plays a role in cell proliferation, apoptosis, and signaling pathways such as NF-κB/MAPK, processes essential for follicular development, oocyte maturation, and implantation [85]. In low-fertility sows, an upregulation of *MIR1307* was reported, indicating that it may negatively regulate target mRNA involved in reproductive functions [86].

Programmed cell death (PCD) is an important biological process which helps to maintain tissue homeostasis, development, and immune regulation [87]. Among the various forms of PCD, apoptosis is the most extensively studied and is necessary for eliminating damaged cells during normal development [88,89]. Excessive apoptosis during early embryogenesis has been identified to be an important contributor to early embryonic mortality in cattle [89]. While necessary for normal development, apoptosis overactivation compromises embryonic viability or fetal development [88,89]. The positional candidate gene for fetal loss in Holstein Heifers, programmed cell death 11 (*PDCD11*), plays an important role in inactivating the activity of p53 to regulate cell growth and turnover [90,91,92]. The expression of this gene with p53 during embryonic development is important in the removal of stem cells that are stressed or damaged to make sure that only healthy cells contribute to the embryo [91,93].

### 3.2. Loci Associated with Fetal Loss in Primiparous Cows

**Figure 2:**
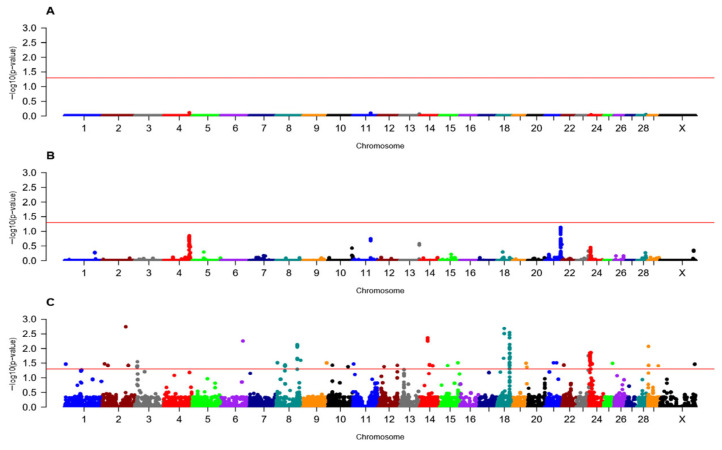
Manhattan plot of genome-wide association analysis for fetal loss in Holstein primiparous cows in additive (**A**), dominant (**B**), and recessive (**C**) inheritance models. The X-axis displays chromosomes by colors, representing the genomic physical positions of each SNP. The Y-axis shows the −log10FDR value for each SNP. The solid red line represents the significance threshold for association (FDR < 0.05) for fetal loss in Holstein primiparous cows. The Y chromosome is not shown since all animals in the study were female.

In primiparous cows, there were 44 loci (142 SNPs) across 18 chromosomes associated (FDR < 0.05) with fetal loss in the recessive model, with the highest number of loci (9) found on BTA24 (Figure 2, Table 2 and Table S2). The loci associated with fetal loss were identified on chromosomes 1, 2, 3, 6, 8, 9, 10, 11, 12, 14, 15, 18, 19, 21, 22, 23, 24, 25, 29, and X (Table 2). The locus on BTA24 harbored the highest number (21) of SNPs (Table 2 and Table S2). No loci were associated with fetal loss in the additive and dominant models (Figure 2; Table 2 and Table S2). Comparable GWAA results were reported by Suarez et al. (2025) [29], where most of the associated loci (FDR < 0.05) with spontaneous abortion in primiparous Holstein cattle bred by AI were identified under the recessive model [28]. The proportion of variance explained by each locus varied from 0.007% and 0.013% (Table 2). There were 87 positional candidate genes associated with fetal loss in the recessive inheritance model. (Table 2). The greatest number of positional candidate genes (10) was identified at locus 24 on BTA18 (Table 2).

In primiparous cows, the three strongest associations were located on BTA2 (FDR = 1.81 × 10^−3^) and BTA18 (FDR = 2.07 × 10^−3^ for locus 22 and FDR = 2.87 × 10^−3^ for locus 25). The locus on chromosome 2 contained only one positional candidate gene, microtubule-associated protein 2 (*MAP2*). No positional candidate genes were located on locus 22 on BTA18; however, nine positional candidate genes (*LYPD5*, *ZNF283*, *IRGC*, *KCNN4*, *SMG9*, *LOC104974883*, *LOC512005*, *LOC526915*, and *LOC616722)* were located within locus 25 on BTA18.

*Map2* is differentially expressed in the brain during gestation in mice [94]. *MAP2* is also critical in the developing dendrites of fetal brains at 21–22 weeks of gestation and continues to be important until six months of gestation in humans [95]. In mice, the expression of *Map2* was observed around embryonic day 14 [96]. In humans, *MAP2* was overexpressed in the fetal neocortex between 16 and 22 weeks of gestation, where its patterns reflect neuronal differentiation and mark specific cell types like Cajal–Retzius and subplate neurons [97]. In cats, *MAP2* was expressed in the first neurons generated in the cortex, located in the marginal zone and subplate during fetal life, where they provide temporary but crucial support for brain development [98]. Based on this, aberrant *MAP2* function or regulation could negatively affect brain development in the fetus and pregnancy success.

Positional candidate genes on BTA18 with functions related to fertility include potassium calcium-activated channel subfamily N member 4 (*KCNN4*) and suppressors with morphogenetic effect on genitalia 9 (*SMG9*). *KCNN4* participates in the relaxation of smooth muscle, in non-pregnant animals and during early- to mid-pregnancy [99]. Another subtype, big-conductance calcium-activated potassium channel (*BKCa*), contributes to vascular relaxation, regulation of uterine muscle activity, and remodeling of uterine arteries during pregnancy [100]. These activities improve placental blood flow, ensuring adequate oxygen and nutrient delivery to the fetus [100]. In addition, Ca^2+^-activated K^+^ channels (*KCa*) broadly regulate uteroplacental blood flow throughout gestation [101].

In Holstein bulls, *SNG9* is a positional candidate gene involved in the rate of embryo cleavage [102], supporting its role in reproductive performance. Functionally, SMG9 is a key component of the SMG1C complex composed of SMG1, SMG8, and SMG9 proteins [103]. The SMG1C complex regulates the nonsense-mediated mRNA decay *CNNM2*, *TAF5*, *ATP5MK*, *MIR1307*, *PDCD11*, and *LOC104975977* pathway, which controls mRNA quality to ensure proper gene expression [102]. SMG1, another subunit of the complex, was found to be indispensable for embryogenesis because its deficiency in mice resulted in embryonic lethality [104]. Moreover, nonsense-mediated mRNA decay was also found to affect embryonic cell fate decisions, implantation, and germ cell and embryonic development [105,106]. In human males, an altered nonsense-mediated mRNA decay function leads to male infertility characterized by testicular degeneration and loss of mature sperm [107].

### 3.3. Loci Associated with Fetal Loss That Are Shared with Production Traits

Loci and their corresponding positional candidate genes associated with fetal loss in Holstein heifers and primiparous cows were compared with production traits (milk yield, milk protein yield, milk fat yield, and milk fatty acid amount) to identify potential correlation and determine whether selection for improved fertility could have adverse effects on production traits. In Holstein heifers, locus 13 on BTA26 at approximately 23 Mb contained two *SNPs* that were previously reported in association with milk production traits (Table S5). This locus contained several positional candidate genes (*CNNM2*, *TAF5*, *ATP5MK*, *MIR1307*, *PDCD11*, and *LOC104975977*) that were reported to be associated with milk fat yield in Nordic Red cattle [108] and with milk fatty acid quantity in Holstein cows [109] (Table S5). In Holstein heifers, the A allele of the SNP *rs136783842* at 23,698,168 bp from locus 13 on BTA26 was associated with an increased risk of fetal loss (it was undesirable) and was also reported to reduce milk fat yield in Nordic Red cattle [108]. For a breeding program, this allele (A) would be undesirable, as it increases the risk of fetal loss and reduces milk fat production. Therefore, selection against this allele could improve reproductive efficiency and production traits. In Holstein heifers, the A allele of the SNP *rs133863660* on BTA26 at 23,993,189 bp, also within locus 13 (D’ = 0.72), increased the risk of fetal loss. This SNP was associated with the quantity of milk fatty acid in Holstein cows [109] (Table S5). However, Gebreyesus et al. (2019) [109] did not mention which allele increased milk fatty acid amount, making it unclear whether selecting against this allele to reduce fetal loss would also decrease milk fatty acid content in Holstein heifers.

In primiparous cows, loci and their positional candidate genes associated with fetal loss were not shared with production traits such as milk yield, milk protein yield, milk fat yield, and milk fatty acid quantity. This suggests that simultaneous selection for reduced fetal loss and improved production traits is possible without compromising genetic progress in production traits in primiparous cows. Similar results were reported by Suarez et al. (2025) [29], where loci and positional candidate genes associated with spontaneous abortion in primiparous Holstein cattle were not shared in previous production studies. This genetic independence provides an opportunity to integrate reproductive traits such as reduced fetal loss into genomic selection indices without risking unfavorable effects on milk yield or composition and help to reduce economic losses linked to reproductive failure.

The sharing of loci associated with traits related to milk production and fetal loss is consistent with some reports but is contradictory to others. If the favorable alleles for fetal loss are the unfavorable alleles for production traits, then there are potential genetic trade-offs between reproductive success and high production that should be weighed in multi-trait selection indices [110,111,112]. An allele (A) which was linked to an increased risk of fetal loss in Holstein heifers in this study was also associated with lower milk fat yield in Nordic Red cattle [108], suggesting a pleiotropic effect or LD with genes that influence both reproductive success and milk production traits. A genetic correlation between fertility and milk production traits has been reported in Holstein-Friesian, Brown Swiss, Simmental [113], and New Zealand dairy cattle [114,115]. These genetic relationships between milk production and fertility traits may complicate their simultaneous improvement in dairy cattle [115]. These results highlight the need to balance reproductive and production traits in genomic selection strategies and to identify causal mutations responsible for these traits to aid in selection.

### 3.4. Recessive Inheritance of Fetal Loss

In this study, loci associated with fetal loss in both Holstein heifers and primiparous cows were identified with the recessive inheritance model, while no associations were observed under additive or dominant models. These results are consistent with those reported by Suarez et al. (2024, 2025) [28,29], where the majority of loci associated with spontaneous abortion in Holstein heifers and primiparous cows were identified under the recessive model. The recurrence of this recessive genetic inheritance pattern across different independent populations suggests that deleterious recessive alleles play an important role in causing pregnancy losses in dairy cattle populations.

In cattle, increased homozygosity and deleterious recessive alleles have been linked to reduced fertility, calving difficulties, and embryonic mortality [33,34,35], and recent reviews highlight the strong impact of non-additive effects, including homozygous deleterious alleles, on reproductive performance [36].

### 3.5. Estimated Heritability of Fetal Loss

Heritability of fetal loss in Holstein heifers was near zero (0.01 ± 0.009), which was expected since all associated loci (FDR < 0.05) were recessive, and heritability captures only additive effects. The estimated heritability for fetal loss in primiparous cows was 0 ± 0.012. This near-zero estimate may be explained by the absence of associations (FDR < 0.05) detected under the additive model (Figure 2 and Table 2). Loci associated with fetal loss under the recessive model would not contribute to the estimate of heritability because it reflects additive genetic variance. These low estimates for heritability for fetal loss align with previous reports for spontaneous abortion in primiparous cows: 0.09 (±0.08) by Oliver et al. (2019) [12] and 0.03 (±0.053) in primiparous cows bred by AI reported by Suarez et al. (2025) [29]. Heritability estimates for fertility in cattle are generally low (0.015–0.18) [116,117,118,119,120], and our results are consistent with these findings. The low heritability observed reflects a genetic architecture dominated by non-additive effects, including recessive deleterious alleles and inbreeding depression, rather than a lack of genetic influence [12,28,29]. As the association of fetal loss is shown with the recessive inheritance model, it also emphasizes that inbreeding of cattle will further result in decreased fertility.

### 3.6. Comparison of Loci Associated with Fetal Loss in Heifers and Primiparous Cows

This study enhanced understanding of the genetic basis of fetal loss in Holstein heifers and primiparous cows by identifying loci and positional candidate genes associated with fetal loss. No loci associated with fetal loss were shared between heifers and primiparous cows. The difference in loci associated with fetal loss in different parities could be due to several factors. One explanation could be the inherent bias introduced by culling that occurs when heifers abort, as their genetics are not represented within the primiparous cow populations. A second explanation could be that the physiological demands placed on a primiparous cow (who is growing, lactating, and recovering from parturition) differ from those placed on a heifer (who is growing), which impacts the loci associated with fetal loss. Third, the lack of shared loci could point to a lack of sufficient power to detect all associations with fetal loss in the two different parities of cattle. It is likely that all three factors are involved to some degree.

### 3.7. Comparison of Loci Associated with Fetal Loss and Other Fertility Traits

The sharing of loci associated with fetal loss and those reported in previous fertility research confirms their role in fertility-related traits and provides support for their use in genomic selection. When comparing the loci identified as associated with fetal loss in Holstein heifers for other fertility traits, four loci (4, 7, 10, and 12) were shared (Table S3). The first locus (4) was on BTA3 at 115 Mb (Table S3). This locus was previously identified as associated with heifer conception rate at first service and the number of times bred by AI before achieving pregnancy in two separate GWAA studies [56,70] (Table S3). A second locus (7) on BTA17 at 15 Mb, identified as associated with fetal loss in the current study, was associated with daughter pregnancy rate in a study by Cole and colleagues (2011) [72] and spontaneous abortion (SA) in Holstein cattle [29] (Table S3). Additionally, loci 10 and 12 on BTA25, located at 37 Mb and 39 Mb, respectively, were previously reported to be associated with spontaneous abortion (SA) in Holstein heifers by Suarez et al. (2024) [28] and with conception rate (CR) in American Braford, Brangus, and Simbrah cattle by McDaneld et al. (2014) [71] (Table S3).

For primiparous cows, four genomic regions (locus 14, 17, 24 and 31) were previously reported as fertility-associated loci (Table S4). The first locus, located on BTA11 at 26 Mb, corresponds to a locus associated with conception rate in Jersey cattle as reported by Rezende et al. (2018) [121]. The second locus, on BTA14 at 42 Mb (locus 17), overlaps with a locus associated with the number of times bred by AI before achieving pregnancy in US Holstein heifers [56] (Table S4). The third shared locus on BTA18 at 52 Mb (locus 24) has been associated with daughter pregnancy rate and length of productive life in previous studies [72,122]. Within this region, several candidate genes have been identified, including *PHLDB3*, *ETHE1*, *ZNF575*, *XRCC1*, and *PINLYP* (Table S4). Finally, the fourth shared locus, located on BTA22 at 7 Mb (locus 31), was shared with loci associated with conception rate and pregnancy rate in Holstein cattle [69]. Positional candidate genes in this region include *CRTAP*, *SUSD5*, and *FBXL2* (Table S4).

## 4. Conclusions

This study confirms that genetic factors contribute to fetal loss in dairy cattle and reveals that these effects differ by parity, providing new insight into the complexity of pregnancy maintenance across reproductive stages. The findings highlight the importance of considering parity in fertility improvement and genomic selection strategies. Further research is needed to clarify the biological mechanisms underlying these parity-specific effects and to explore their broader implications for improving reproductive success in dairy cattle.

## Figures and Tables

**Table 1:** Loci and positional candidate genes associated with fetal loss in Holstein heifers with FDR < 0.05.

BTA ^1^	Locus ^2^	SNP Count ^3^	FDR ^4^	PVE (%) ^5^	Positional Candidate Genes for Locus ^6^
1	1	1	4.98 × 10^−2^	0.003	
1	2	3	3.83 × 10^−2^	0.0037	*SLC19A1*, *LOC100849587*, *PCBP3*
2	3	1	1.96 × 10^−3^	0.0051	
3	4	2	1.52 × 10^−3^	0.005	
7	5	3	1.96 × 10^−3^	0.004	*LOC100140613*
14	6	3	1.59 × 10^−3^	0.004	
17	7	1	3.80 × 10^−3^	0.004	
24	8	1	5.30 × 10^−3^	0.004	*BCL2*
25	9	1	4.21 × 10^−2^	0.003	
25	10	9	5.80 × 10^−4^	0.005	*TMEM225B*, *LOC112444278*, *ZNF655*, *ZNF789*
25	11	1	2.41 × 10^−2^	0.004	*OCM*, *LOC100850875*, *CCZ1*, *RSPH10B*
25	12	1	1.62 × 10^−2^	0.004	*MMD2*, *RADIL*
26	13	2	1.44 × 10^−3^	0.005	*CNNM2*, *TAF5*, *ATP5MK*, *MIR1307*, *PDCD11*, *LOC104975977*
27	14	3	1.67 × 10^−3^	0.005	
27	15	3	1.34 × 10^−3^	0.005	*LOC112444679*, *LOC107131907*, *LOC781220*
X	16	6	1.66 × 10^−3^	0.004	*MORF4L2*, *LOC101901997*, *GLRA4*

^1^ *Bos taurus* chromosome with association with fetal loss. ^2^ Locus associated with fetal loss. ^3^ Number of associated SNPs defining the locus. ^4^ False discovery rate (FDR)-adjusted *p* values for locus associated with fetal loss. ^5^ The proportion of variance explained by the locus. ^6^ Positional candidate genes located within 29 kb upstream or downstream of the SNPs associated with fetal loss based on the NCBI database and the cow genome assembly ARS-UCD2.0 (https://www.ncbi.nlm.nih.gov/datasets/genome/GCF_002263795.3/) ((accessed on 6 June 2025)). Positional candidate gene functional information was obtained from NCBI (https://www.ncbi.nlm.nih.gov/) and Ensembl (https://useast.ensembl.org/Bos_taurus/Info/Index) ((accessed on 6 June 2025)).

**Table 2:** Loci and positional candidate genes associated with fetal loss in Holstein primiparous cows with FDR < 0.05.

BTA ^1^	Locus ^2^	SNP Count ^3^	FDR ^4^	PVE (%) ^5^	Positional Candidate Genes for Locus ^6^
1	1	2	3.43 × 10^−2^	0.007	*SCAF4*, *SOD1*, *TRNAG-CCC*
2	2	1	3.36 × 10^−2^	0.007	*GULP1*
2	3	3	3.77 × 10^−2^	0.007	*SCRN3*, *CIR1*, *LOC112443637*
2	4	1	1.81 × 10^−3^	0.013	*MAP2*
2	5	3	3.82 × 10^−2^	0.007	
3	6	1	2.85 × 10^−2^	0.008	*TRNAG-UCC*, *OR10J30P*, *APCS*
6	7	1	5.54 × 10^−3^	0.01	
8	8	2	3.03 × 10^−2^	0.007	*GALNTL6*
8	9	15	3.65 × 10^−2^	0.007	*LOC132345943*
8	10	12	7.33 × 10^−3^	0.01	*SYK*
8	11	1	2.53 × 10^−2^	0.008	*ECPAS*
9	12	2	3.08 × 10^−2^	0.007	*PRKN*
10	13	1	3.71 × 10^−2^	0.007	
11	14	1	3.39 × 10^−2^	0.007	*KANSL3*, *FER1L5*
12	15	1	3.76 × 10^−2^	0.007	*FARP1*, *LOC112449164*
14	16	3	4.34 × 10^−3^	0.01	*CPA6*
14	17	6	3.56 × 10^−2^	0.007	
14	18	4	3.86 × 10^−2^	0.007	
15	19	1	3.82 × 10^−2^	0.007	*LOC101903557*, *LOC100848689*, *MIR125B-1*
15	20	1	3.07 × 10^−2^	0.008	*LOC132342364*
18	21	1	2.17 × 10^−2^	0.008	
18	22	2	2.07 × 10^−3^	0.012	
18	23	1	3.26 × 10^−2^	0.007	*ARHGEF1*, *CD79A*, *RPS19*, *DMRTC2*, *LYPD4*
18	24	11	2.04 × 10^−2^	0.008	*PHLDB3*, *ETHE1*, *XRCC1*, *PINLYP*, *IRGQ*, *ZNF575*, *SRRM5*, *ZNF428*, *CADM4*, *PLAUR*
18	25	11	2.87 × 10^−3^	0.011	*LYPD5*, *ZNF283*, *IRGC*, *KCNN4*, *SMG9*, *LOC104974883*, *LOC512005*, *LOC526915*, *LOC616722*
18	26	6	9.57 × 10^−3^	0.009	*ZNF226*, *ZNF227*, *ZNF233*
18	27	2	1.97 × 10^−2^	0.008	*NOVA2*, *LOC112442342*
19	28	1	3.18 × 10^−2^	0.007	*RNF157*, *FOXJ1*
21	29	1	3.08 × 10^−2^	0.007	*STXBP6*
21	30	1	3.06 × 10^−2^	0.008	*TTC6*
22	31	3	3.65 × 10^−2^	0.007	*CRTAP*, *SUSD5*, *FBXL2*, *LOC112443433*
23	32	1	1.77 × 10^−2^	0.008	*LYRM4*, *PPP1R3G*
24	33	21	1.43 × 10^−2^	0.008	*ZNF407*
24	34	1	3.76 × 10^−2^	0.007	*DIPK1C*, *C24H18orf63*, *SPACDR*
24	35	1	2.89 × 10^−2^	0.008	
24	36	1	3.09 × 10^−2^	0.008	
24	37	1	3.05 × 10^−2^	0.008	
24	38	1	1.42 × 10^−2^	0.008	*RTTN*
24	39	1	1.36 × 10^−2^	0.008	*CD226*
24	39	1	1.39 × 10^−2^	0.008	*CD226*
24	40	4	3.31 × 10^−2^	0.007	*SERPINB8*, *TRNAK-UUU*, *LOC112444149*
25	41	1	3.20 × 10^−2^	0.007	*NYAP1*, *TSC22D4*, *SPACDR*, *PPP1R35*, *MEPCE*, *ZCWPW1*
29	42	3	8.52 × 10^−3^	0.01	*FAT3*, *LOC112444945*
29	43	1	3.89 × 10^−2^	0.007	*MEN1*, *TRNAE-CUC*, *CDC42BPG*, *EHD1*
X	44	2	3.42 × 10^−2^	0.007	*LOC101902122*, *TRNAC-ACA*

^1^ *Bos taurus* chromosome with association with fetal loss. ^2^ Locus associated with fetal loss. ^3^ Number of associated SNPs defining the locus. ^4^ False discovery rate (FDR)-adjusted *p* values for loci associated with fetal loss. ^5^ The proportion of variance explained by the locus. ^6^ Positional candidate genes located within 29 kb upstream or downstream of the SNPs inside the locus associated with fetal loss based on the NCBI database and the cow genome assembly ARS-UCD2.0 (https://www.ncbi.nlm.nih.gov/datasets/genome/GCF_002263795.3/) (accessed 6 June 2025). Positional candidate gene functional information was obtained from NCBI (https://www.ncbi.nlm.nih.gov/) and Ensembl (https://useast.ensembl.org/Bos_taurus/Info/Index accessed on 6 June 2025).

## Data Availability

The datasets presented in this study can be found in online repositories. Data generated and/or analyzed during the current study are pending review and acceptance by the USDA Ag Data Commons (https://figshare.com/s/a6978c716b25f1562f1a (accessed on 3 June 2025)).

## Supplementary Materials

The following supporting information can be downloaded at https://www.mdpi.com/article/10.3390/ani16020293/s1. Figure S1. Plots A and B show the principal component analysis (PCA) of the first two principal components (PC1 and PC2), representing the greatest genetic variance in the primiparous cow and heifer populations, respectively. Table S1. Loci associated with fetal loss in Holstein heifers. Table S2. Loci associated with fetal loss in primiparous cows. Table S3. Loci associated with fetal loss in Holstein heifers that were shared in previous fertility studies. Table S4. Loci associated with fetal loss in primiparous cows that were shared in previous fertility studies. Table S5. Loci associated with fetal loss in Holstein heifers and primiparous cows that were shared in previous production trait studies.

## Abbreviations

The following abbreviations are used in this manuscript:

AI	Artificial insemination
AI-REML	Average information restricted maximum likelihood
ANOVA	Analysis of variance
Bp	Base pairs
BTA	*Bos taurus* chromosome
CR	Conception rate
D’	Standardized disequilibrium coefficient
DPR	Daughter pregnancy rate
EMMAX	Efficient mixed model association eXpedited
FDR	False discovery rate
GBLUP	Genomic linear unbiased predictor
GWAA	Genome-wide association analysis
HCR1	Heifer conception rate at first service
Kb	Kilobase
LD	Linkage disequilibrium
LPL	Length of productive life
Mb	Megabase
*p*	*p* value
PCG	Positional candidate gene
Pos	Position
PR	Pregnancy rate
PVE	Proportion of variance explained
QTL	Quantitative trait locus
SNP	Single-nucleotide polymorphism
SA	Spontaneous abortion or fetal loss
SVS	SNP and variation suite
TBRD	Number of times bred by artificial insemination before a pregnancy was achieved
λ_GC_	Genomic inflation factor
