# Supplementary material for: Identifying the Genetic Basis of Fetal Loss in Cows and Heifers Through a Genome-Wide Association Analysis
Source: Animals (Basel). 2026 Jan 17;16(2):293. doi: 10.3390/ani16020293 (PMC12837429; doi:10.3390/ani16020293)
Supplement: Supplementary file 1 [file animals-16-00293-s001.zip › animals-4037489-supplementary.pdf]

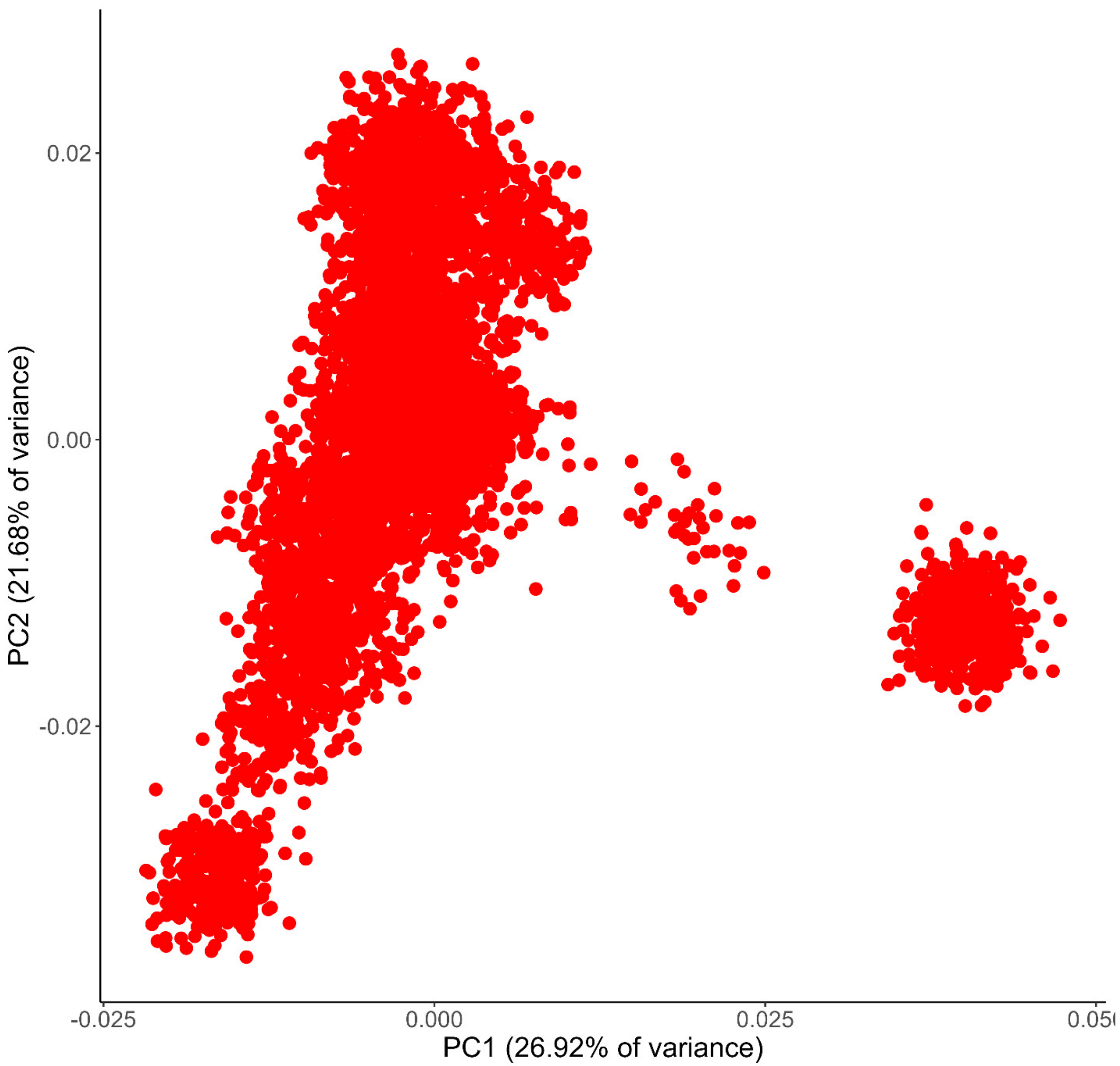

0.02
0.00
-0.02
-0.025
0.000
0.025
PC1 (26.92% of variance)
PC2 (21.68% of variance)

A. Holstein Heifer principal component analysis

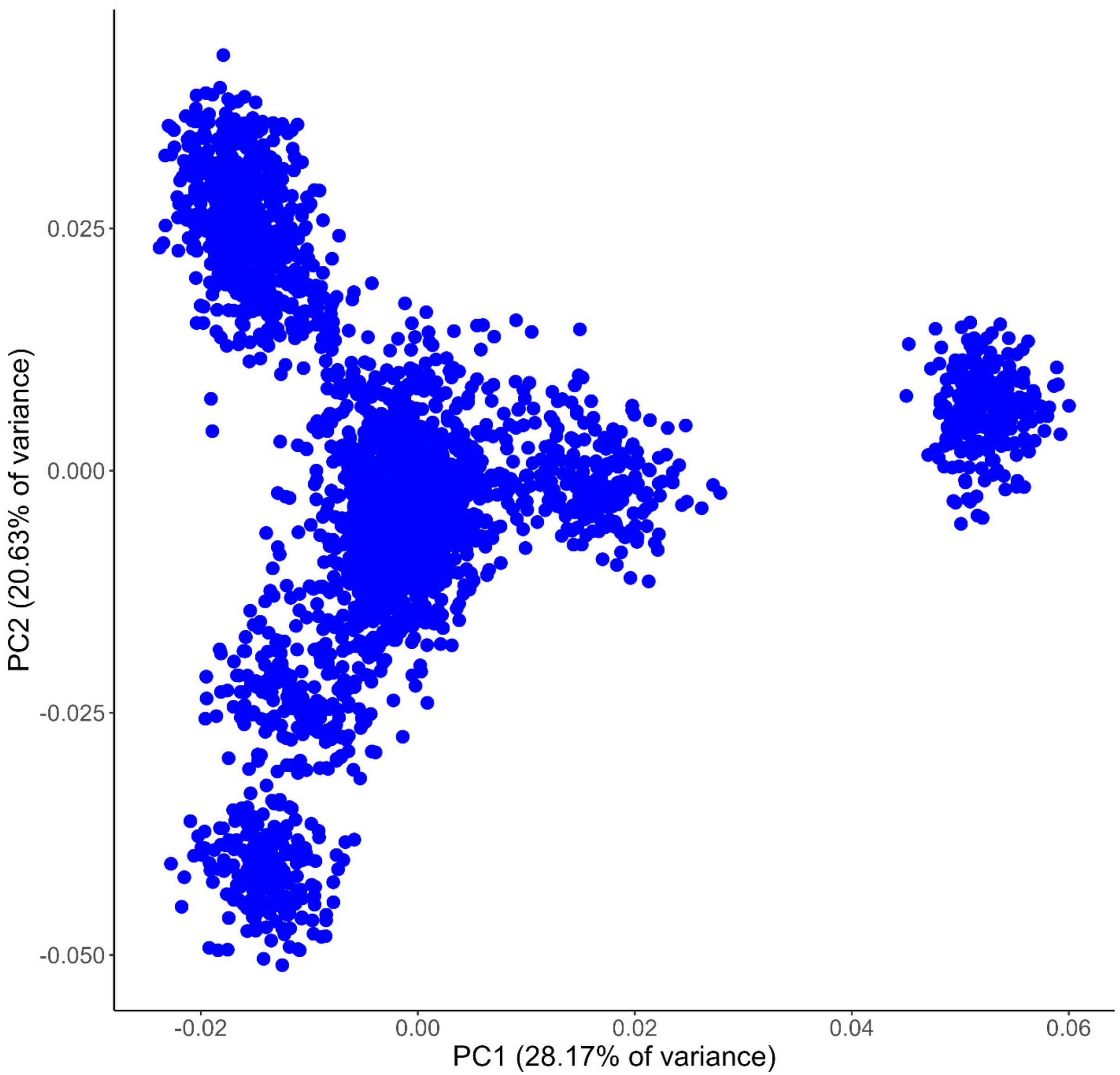

PC2 (20.63% of variance)
0.025
0.000
-0.025
-0.050
-0.02
0.00
0.02
0.04
0.06
PC1 (28.17% of variance)

B. Primiparous cow principal component analysis

Figure S1. Plots A and B show the principal component analysis (PCA) of the first two principal components (PC1 and PC2), representing the greatest genetic variance in the primiparous cow and heifer populations, respectively.

Table S1: Loci associated with fetal loss in Holstein heifers.

BTA[1]	Locus[2]	Pos (Mb)[3]	FDR[4]	PVE (%)[5]	PCG[6]
1	1	138.8	4.98×10^{-2}	0.003	
1	2	147.0	4.05×10^{-2}	0.003	*SLC19A1, LOC100849587*
1		147.1	3.93×10^{-2}	0.003	*LOC100849587, PCBP3*
1		147.1	3.83×10^{-2}	0.003	*LOC100849587, PCBP3*
2	3	98.7	1.96×10^{-3}	0.005	
3	4	115.0	1.74×10^{-3}	0.005	
3		115.0	1.52×10^{-3}	0.005	
7	5	32.4	2.14×10^{-3}	0.004	
7		33.4	1.96×10^{-3}	0.004	*LOC100140613*
14	6	13.4	1.77×10^{-3}	0.004	
17	7	15.0	3.80×10^{-3}	0.004	
24	8	61.9	5.30×10^{-3}	0.004	*BCL2*
25	9	24.04	4.21×10^{-2}	0.003	
25	10	37.2	3.94×10^{-2}	0.003	
25		37.3	2.32×10^{-3}	0.005	*TMEM225B, LOC112444278, ZNF655, ZNF789*
25		37.4	5.80×10^{-4}	0.005	
25	11	38.5	2.41×10^{-3}	0.004	*OCM, LOC100850875, CCZ1, RSPH10B*
25	12	39.6	1.62×10^{-2}	0.004	*MMD2, RADIL*
26	13	23.9	1.72×10^{-3}	0.005	*CNNM2*

26		24.2	1.44×10^{-3}	0.005	*TAF5, ATP5MK, MIR1307, PDCD11, LOC104975977*
27	14	17.9	2.01×10^{-3}	0.005	
27	15	17.9	1.55×10^{-3}	0.005	*LOC112444679*
27		18.0	1.43×10^{-3}	0.005	*LOC112444679, LOC107131907*
27		18.0	1.34×10^{-3}	0.005	*LOC107131907, LOC781220*
X	16	57.7	3.48×10^{-2}	0.003	*MORF4L2, LOC101901997, GLRA4*
X		57.7	1.77×10^{-3}	0.004	*MORF4L2, LOC101901997, GLRA4*
X		57.7	5.00×10^{-2}	0.003	*MORF4L2, LOC101901997, GLRA4*
X		57.7	4.87×10^{-2}	0.003	*LOC101901997, GLRA4*
X		57.7	1.66×10^{-3}	0.004	*LOC101901997, GLRA4*

[1]*Bos taurus* chromosome. [2]Sequentially numbered loci associated with fetal loss from BTA1 to BTAX along with their associated single nucleotide polymorphism (SNPs). The horizontal line across the table delineates each locus. [3]Genomic locations of associated SNPs, given in megabase (Mb), based on nucleotide numbering in the ARS-UCD 1.2 reference genome (https://www.ncbi.nlm.nih.gov/datasets/genome/GCF_002263795.1/ (6 June 2025)). [4]False discovery rate (FDR) adjusted P values for SNPs associated with fetal loss. [5]Proportion of variance explained (PVE) by each SNP associated with fetal loss. [6]Positional candidate genes located within 29 kb upstream or downstream of the SNPs associated with fetal loss based on the NCBI database and the cow genome assembly ARS-UCD2.0 (https://www.ncbi.nlm.nih.gov/datasets/genome/GCF_002263795.3/ (6 June 2025)). Positional candidate genes functional information was obtained from NCBI (https://www.ncbi.nlm.nih.gov/ (6 June 2025)) and Ensembl (https://useast.ensembl.org/Bos_taurus/Info/Index (6 June 2025)).

Table S2: Loci associated with fetal loss in primiparous cows.

BTA[1]	Locus[2]	Pos (Mb)[3]	FDR[4]	PVE[5]	PCG[6]
1	1	3.1	3.43×10^{-2}	0.007	*SCAF4, SOD1, TRNAG-CCC*
2	2	8.0	3.36×10^{-2}	0.007	*GULP1*
2	3	22.4	3.85×10^{-2}	0.007	*SCRN3, CIR1*

2		22.5	3.77×10^{-2}	0.007	*LOC112443637*
2	4	97.9	1.81×10^{-3}	0.013	*MAP2*
2	5	108.4	3.82×10^{-2}	0.007	
3	6	10.2	2.85×10^{-2}	0.008	*TRNAG-UCC, OR10J30P, APCS*
6	7	91.3	5.54×10^{-3}	0.01	
8	8	4.6	3.07×10^{-2}	0.007	*GALNTL6*
8	9	36.9	4.08×10^{-2}	0.007	*LOC132345943*
8		37.0	3.65×10^{-2}	0.007	*LOC132345943*
8	10	88.2	8.24×10^{-3}	0.01	
8		88.3	2.35×10^{-2}	0.008	
8		88.4	2.31×10^{-2}	0.008	*SYK*
8		88.4	8.33×10^{-3}	0.01	*SYK*
8	11	102.5	2.53×10^{-2}	0.008	*ECPAS*
9	12	99.1	3.12×10^{-2}	0.007	*PRKN*
9		99.1	3.08×10^{-2}	0.007	*PRKN*
10	13	17.2	3.71×10^{-2}	0.007	
11	14	2.6	3.39×10^{-2}	0.007	*KANSL3, FER1L5*
12	15	79.3	3.76×10^{-2}	0.007	*FARP1, LOC112449164*
14	16	33.7	5.58×10^{-3}	0.01	*CPA6*
14		33.8	4.88×10^{-3}	0.01	*CPA6*
14		33.8	4.34×10^{-3}	0.01	*CPA6*
14	17	41.8	3.74×10^{-2}	0.007	
14	18	54.5	3.96×10^{-2}	0.007	
15	20	33.3	3.82×10^{-2}	0.007	*LOC101903557, LOC100848689, MIR125B-1*
15	19	76.2	3.07×10^{-2}	0.008	*LOC132342364*
18	21	27.0	2.17×10^{-2}	0.008	
18	22	29.9	3.10×10^{-2}	0.012	
18	23	51.7	3.26×10^{-2}	0.007	*ARHGEF1, CD79A, RPS19, DMRTC2, LYPD4*
18	24	52.0	2.28×10^{-2}	0.008	*PHLDB3, ETHE1, ZNF575, XRCC1, PINLYP*
18		52.0	2.25×10^{-2}	0.008	*ETHE1, ZNF575, XRCC1, PINLYP*

18		52.1	2.14×10^{-2}	0.008	*XRCC1, PINLYP*
18		52.1	2.11×10^{-2}	0.008	
18		52.1	2.07×10^{-2}	0.008	*XRCC1, PINLYP, IRGQ*
18		52.1	2.04×10^{-2}	0.008	*PINLYP, IRGQ, ZNF576, ZNF428, SRRM5*
18		52.1	2.99×10^{-2}	0.008	*IRGQ, ZNF576, ZNF428, SRRM5, CADM4, PLAUR*
18	25	52.2	3.74×10^{-2}	0.007	*LOC512005, LOC526915, IRGC, LOC616722*
18		52.2	3.83×10^{-2}	0.007	*LOC616722, SMG9, LOC104974883, KCNN4*
18		52.3	3.80×10^{-2}	0.007	*SMG9, LOC104974883, KCNN4*
18		52.3	3.77×10^{-2}	0.007	*SMG9, LOC104974883, KCNN4, LYPD5*
18		52.3	4.30×10^{-3}	0.011	*SMG9, LOC104974883, KCNN4, LYPD5*
18		52.3	2.87×10^{-3}	0.011	*LYPD5, ZNF283*
18		52.3	7.27×10^{-3}	0.01	*ZNF283*
18	26	52.4	1.48×10^{-2}	0.008	*ZNF226, ZNF227, ZNF233*
18		52.5	3.74×10^{-2}	0.007	*ZNF227, ZNF233*
18	27	53.8	1.97×10^{-2}	0.008	*NOVA2, LOC112442342*
18		53.8	1.93×10^{-2}	0.008	*NOVA2, LOC112442342*
19	28	56.1	3.18×10^{-2}	0.007	*RNF157, FOXJ1*
21	29	35.5	3.08×10^{-2}	0.007	*STXBP6*
21	30	48.4	3.06×10^{-2}	0.008	*TTC6*
22	31	7.5	3.72×10^{-2}	0.007	*CRTAP, SUSD5*
22		7.6	3.68×10^{-2}	0.007	*SUSD5, FBXL2, LOC112443433*
22		7.6	3.65×10^{-2}	0.007	*SUSD5, FBXL2, LOC112443433*
23	32	49.3	1.77×10^{-2}	0.008	*LYRM4, PPP1R3G*
24	33	3.8	2.67×10^{-2}	0.008	*ZNF407*
24	34	4.3	3.76×10^{-2}	0.007	*DIPK1C, C24H18orf63, SPACDR*
24	35	5.7	2.89×10^{-2}	0.008	
24	36	5.8	3.09×10^{-2}	0.008	
24	37	6.1	3.05×10^{-2}	0.008	
24	38	7.4	1.42×10^{-2}	0.008	*RTTN*
24	39	7.6	1.39×10^{-2}	0.008	*CD226*

24		7.6	1.36 x 10^{-2}	0.008	*CD226*
24	40	12.6	3.42 x 10^{-2}	0.007	*SERPINB8, TRNAK-UUU, LOC112444149*
25	41	36.6	3.20 x 10^{-2}	0.007	*NYAP1, TSC22D4, SPACDR, PPP1R35, MEPCE, ZCWPW1*
29	42	2.1	8.53 x 10^{-2}	0.01	*FAT3*
29		2.5	3.75 x 10^{-2}	0.007	*FAT3, LOC112444945*
29	43	43.6	3.89 x 10^{-2}	0.007	*MEN1, TRNAE-CUC, CDC42BPG, EHD1*
X	44	145.6	3.46 x 10^{-2}	0.007	
X		145.8	3.42 x 10^{-2}	0.007	*LOC101902122, TRNAC-ACA*

[1]*Bos taurus* chromosome. [2]Sequentially numbered loci from BTA1 to BTAX associated fetal loss, along with their corresponding single nucleotide polymorphism (SNPs). The horizontal line across the table delineates each locus. [3]Genomic locations of associated SNPs, given in megabase (Mb), based on nucleotide numbering in the ARS-UCD 1.2 reference genome (https://www.ncbi.nlm.nih.gov/datasets/genome/GCF_002263795.1/ (6 June 2025)). [4]False discovery rate (FDR) adjusted P values for SNPs associated with fetal loss. [5]Proportion of variance explained (PVE) by each SNP associated with fetal loss. [6]Positional candidate genes located within 29 kb upstream or downstream of the SNPs associated with fetal loss based on the NCBI database and the cow genome assembly ARS-UCD2.0 (https://www.ncbi.nlm.nih.gov/datasets/genome/GCF_002263795.3/ (6 June 2025)). Positional candidate genes functional information was obtained from NCBI (https://www.ncbi.nlm.nih.gov/) and Ensembl (https://useast.ensembl.org/Bos_taurus/Info/Index (6 June 2025)).

Table S3. Loci associated with fetal loss in Holstein heifers that were shared in previous fertility studies.

BTA[1]	Locus[2]	Pos (Mb)[3]	FDR[4]	PVE (%)[5]	PCG[6]	Study[7]	Breed[8]	Trait[9]
3	4	115.0	1.74 x 10^{-3}	0.005		Kiser et al. 2019 [59]	Holstein	HCR1
3	4	115.0	1.74 x 10^{-3}	0.005		Kiser et al. 2019 [59]	Holstein	TBRD
3	4	115.0	1.74 x 10^{-3}	0.005		Galliou et al. 2020 [47]	Holstein	TBRD

3	4	115.0	1.52 x 10^{-3}	0.005		Kiser et al. 2019 [59]	Holstein	HCR1
3	4	115.0	1.52 x 10^{-3}	0.005		Kiser et al. 2019 [59]	Holstein	TBRD
3	4	115.0	1.52 x 10^{-3}	0.005		Galliou et al. 2020 [47]	Holstein	TBRD
17	7	15.0	3.80 x 10^{-3}	0.004		Cole et al. 2011 [61]	Holstein	DPR
17	7	15.0	3.80 x 10^{-3}	0.004		Cole et al. 2011 [61]	Holstein	DPR
17	7	15.0	3.80 x 10^{-3}	0.004		Suarez et al. 2024 [30]	Holstein	SA
25	10	37.2	3.94 x 10^{-2}	0.003		Suarez et al. 2024 [30]	Holstein	SA
25	12	39.6	1.62 x 10^{-2}	0.004	*MMD2, RADIL*	McDaneld et al. 2014 [60]	Braford, Brangus, Simbrah	CR

[1]*Bos taurus* chromosome. [2]Sequentially numbered loci from BTA1 to BTAX associated fetal loss, along with their corresponding single nucleotide polymorphism (SNPs). [3]Genomic locations of associated SNPs, given in megabase (Mb), based on nucleotide numbering in the ARS-UCD 1.2 reference genome (https://www.ncbi.nlm.nih.gov/datasets/genome/GCF_002263795.1/ (6 J une 2 025)). [4]False d iscovery r ate (FDR) adjusted P values for SNPs associated with fetal loss. [5]Proportion of variance explained (PVE) by each SNP associated with fetal loss. [6]Positional candidate genes located within 29 kb upstream or downstream of the SNPs associated with fetal loss based on the NCBI database and the cow genome assembly ARS-UCD2.0 (https://www.ncbi.nlm.nih.gov/datasets/genome/GCF_002263795.3/ (6 J une 2 025)). [7]Previous f ertility s tudies. [8]Cattle breed evaluated in previous study. [9]Fertility trait associated in a previous study as defined by: HCR1 (heifer conception rate at first service), TBRD (number of times bred by artificial insemination before a pregnancy was achieved), DPR (daughter pregnancy rate), SA (spontaneous abortion or fetal loss) and CR (conception rate). Positional candidate genes functional information was obtained from NCBI (https://www.ncbi.nlm.nih.gov/ (6 June 2025)) and Ensembl (https://useast.ensembl.org/Bos_taurus/Info/Index (6 June 2025)).

Table S4. Loci associated with fetal loss in primiparous cows that were shared in previous fertility studies.

BTA[1]	Locus[2]	Pos[3] (Mb)	FDR[4]	PVE (%)[5]	PCG[6]	Study[7]	Breed[8]	Trait[9]
11	14	2.6	3.39×10^{-2}	0.007	*KANSL3, FER1L5*	Rezende et al. 2018 [106]	Jersey	CR
14	17	41.8	3.74×10^{-2}	0.007		Galliou et al. 2020 [47]	Holstein	TBRD
14	17	41.8	3.70×10^{-2}	0.007		Galliou et al. 2020 [47]	Holstein	TBRD
14	17	41.8	3.67×10^{-2}	0.007		Galliou et al. 2020 [47]	Holstein	TBRD
18	24	52.0	2.28×10^{-2}	0.008	*PHLDB3, ETHE1, ZNF575, XRCC1, PINLYP*	Cole et al. 2011 [61]	Holstein	DPR
18	24	52.0	2.28×10^{-2}	0.008	*PHLDB3, ETHE1, ZNF575, XRCC1, PINLYP*	Nayeri et al. 2017 [107]	Holstein	LPL
18	24	52.0	2.25×10^{-2}	0.008	*ETHE1, ZNF575, XRCC1, PINLYP*	Cole et al. 2011 [61]	Holstein	DPR
18	24	52.0	2.21×10^{-2}	0.008	*ETHE1, ZNF575, XRCC1, PINLYP*	Cole et al. 2011 [61]	Holstein	DPR
22	31	7.5	3.72×10^{-2}	0.007	*CRTAP, SUSD5*	Parker Gaddis et al. 2016 [58]	Holstein	CR
22	31	7.6	3.68×10^{-2}	0.007	*SUSD5, FBXL2, LOC112443433*	Parker Gaddis et al. 2016 [58]	Holstein	PR
22	31	7.6	3.65×10^{-2}	0.007	*SUSD5, FBXL2, LOC112443433*	Parker Gaddis et al. 2016 [58]	Holstein	CR

[1]*Bos taurus* chromosome. [2]Sequentially numbered loci from BTA1 to BTAX that were associated with fetal loss, along with their corresponding single nucleotide polymorphism (SNPs). [3]Genomic locations of associated SNPs, given in megabase (Mb), based on nucleotide numbering in the ARS-UCD 1.2 reference genome (https://www.ncbi.nlm.nih.gov/datasets/genome/GCF_002263795.1/ (6 June 2025)). [4]False discovery rate (FDR) adjusted P values for SNPs associated with fetal loss. [5]Proportion of variance explained (PVE) for each SNP associated with fetal loss. [6]Positional candidate genes located within 29 kb upstream or downstream of the SNPs associated with fetal loss based on the NCBI database and the cow genome assembly ARS-UCD2.0 (https://www.ncbi.nlm.nih.gov/datasets/genome/GCF_002263795.3/ (6 June 2025)). [7]Previous fertility studies. [8]Cattle breed evaluated in previous studies. [9]Fertility trait associated in previous studies as defined by: CR

(conception rate), TBRD (number of times bred by artificial insemination before a pregnancy was achieved), DPR (daughter pregnancy rate), LPL (length of productive life), and PR (pregnancy rate). Positional candidate genes functional information was obtained from NCBI (https://www.ncbi.nlm.nih.gov/ (6 J une 2 025)) a nd E nsembl (https://useast.ensembl.org/Bos_taurus/Info/Index (6 June 2025)).

Table S5. Loci associated with fetal loss in Holstein heifers and primiparous cows that were shared in previous production trait studies.

BTA[1]	Locus[2]	Location (Mb)[3]	PVE (%)[4]	PCG[5]	Study[6]	Breed[7]	Trait[8]
26	13	23.6	0.005	*CNNM2*	Pedrosa et al. 2021 [97]	Nordic-Red	Milk fat yield
26	13	23.9	0.005	*TAF5, ATP5MK, MIR1307, PDCD11, LOC104975977*	Gebreyesus et al. 2019 [98]	Holstein	Milk fatty acid yield

[1]*Bos taurus* chromosome. [2]Sequentially numbered loci from BTA1 to BTAX associated fetal loss, along with their corresponding single nucleotide polymorphism (SNPs). [3]Genomic locations of associated SNPs, given in megabase (bp), based on nucleotide numbering in the ARS-UCD 1.2 reference genome (https://www.ncbi.nlm.nih.gov/datasets/genome/GCF_002263795.1/ (6 June 2025)). [4]Proportion of variance explained (PVE) by each SNP associated with fetal loss. [5]Positional candidate genes located within 29 kb upstream or downstream of the SNPs associated with fetal loss based on the NCBI database and the cow genome assembly ARS-UCD2.0 (https://www.ncbi.nlm.nih.gov/datasets/genome/GCF_002263795.3/). [6]Previous production trait studies. [7]Cattle breed evaluated in previous study. [8]Production trait associated in the previous study. Positional candidate genes functional information was obtained from NCBI (https://www.ncbi.nlm.nih.gov/ (6 June 2025)) and Ensembl (https://useast.ensembl.org/Bos_taurus/Info/Index (6 June 2025)).
